# Ozone Exposure Induces Metabolic Disorders and NAD+ Depletion Through PARP1 Activation in Spinal Cord Neurons

**DOI:** 10.3389/fmed.2020.617321

**Published:** 2020-12-17

**Authors:** Shulin Ma, Xu Zhao, Cong Zhang, Panpan Sun, Yun Li, Xiaowen Lin, Tao Sun, Zhijian Fu

**Affiliations:** ^1^Department of Pain Management, Shandong Provincial Hospital, Cheeloo College of Medicine, Shandong University, Jinan, China; ^2^Department of Pain Management, Shandong Provincial Hospital Affiliated to Shandong First Medical University, Jinan, China

**Keywords:** ozone, metabolomics, nicotinamide adenine dinucleotide, ATP level, spinal cord neurons

## Abstract

**Background and Objective:** Ozone therapy has shown therapeutic efficacy in different disorders particularly low back pain (LBP). However, ozone therapy has been associated with toxic effects on the respiratory, endocrine, cardiovascular systems as well as nervous system because of its strong oxidizing capacity. Recent studies have reported possible associations between ozone exposure and metabolic disorders, but the findings are controversial and little is known on the mechanisms of action. This study aims to investigate the cytotoxic effects of ozone exposure and possible mechanism of action in the animal model.

**Methods:** Wistar neonate rats with the age of 24 h after birth were sacrificed by cervical dislocation under general anesthesia, then immersed in 75% alcohol and iodophor for 5 min, respectively. The spinal cord was isolated and cut to samples of ~1 mm^3^ and prepared for further experiments. The spinal cord neurons (SCNs) were exposed to ozone at different concentrations and then cultured in 96-well plates with glass bottom for 7 days. The cell viability, ATP levels and the NAD+ concentration were determined and compared between the different experimental groups and the control group.

**Results:** Analyses of the data by non-targeted liquid chromatography-mass spectrometry (LC-MS) analysis determined the metabolic disorder in SCNs following the ozone exposure. Moreover, our assessments showed that ozone exposure resulted in DNA damage, poly (ADP)-ribose polymerase-1 (PARP1) excessive activation, nicotinamide adenine dinucleotide (NAD+) depletion and decrease of ATP level in SCNs. The PARP1 inhibitor can inhibit the cytotoxic effect of ozone to SCNs without inhibiting the activation of AMP-activated protein kinase (AMPK). Our findings revealed that the cytotoxic effects of ozone to SCNs might be mediated by excessive PARP1 activation and subsequent NAD+ depletion. Moreover, using PARP1 inhibitor can protect SCNs from cytotoxic effects of ozone by preventing NAD+ depletion during ozone exposure.

**Conclusion:** Ozone exposure seems to induce metabolic disorders and NAD+ depletion through excessive PARP1 activation in SCNs.

## Introduction

Ozone is an inorganic molecule and allotrope of oxygen with strong oxidizing capacity. It is a highly reactive agent consisting of three oxygen atoms bonded in a V-like shape (1). It occurs both naturally in the atmosphere and as a man-made product by industrial activities (1, 2). Ozone is the major photochemical constitute of polluted air, which induces a dose-dependent oxidative stress in tissues due to its strong capacity to produce free radicals from different interactions including protein oxidation, enzymatic inactivation, lipoperoxidation of cell membranes, DNA destruction, and cell apoptosis (1, 3, 4). Despite of these interactions, ozone has been widely used for different musculoskeletal disorders (5–7). During the last decade, ozone therapy alone or in combination with other modalities, has been extensively used in clinical practice for treatment of herniated discs, low back pain (LBP) and other chronic pains (4–8). Further translational and clinical trials have shown the therapeutic efficacy and safety of ozone for other disorders including degenerative disorders, vascular and immune diseases (4, 9–12). The main administrations routes of ozone in the ozone therapy are percutaneous, intradiscal, and intramuscular routes. Current evidence shows the potential therapeutic efficacy of ozone therapy in herniated discs and for pain management in LBP (4, 6, 13, 14). However, ozone therapy has been associated with toxic effects on the respiratory, endocrine, cardiovascular systems as well as nervous system because of its strong oxidizing property and inducing systemic inflammation (12, 15–19). Ginanneschi et al. reported that transcutaneous intradiscal injection of ozone for L4–L5 disk herniation resulted in ventral and dorsal root injury (20). In this regard, some studies have investigated the effects and mechanisms of action of ozone exposure on metabolic disorders and reported the associations between ozone exposure and metabolic disorders (11, 18). Although many studies have been conducted on the cytotoxic effects of ozone, the underlying signaling pathways and molecular mechanisms of susceptibility and the disorder are not fully understood. However, evidence from human and animal studies suggests that ozone-induced neuroinflammation, oxidative stress, microglial activation, cerebrovascular dysfunction, and alterations in the blood-brain barrier are the main mechanisms of ozone induced cytotoxicity in central nervous system (11). Defining the cytotoxicity and mechanisms of action of ozone exposure particularly in spinal cord neurons (SCNs) is necessary to develop efficient ozone therapy as well as new protective strategies for individuals at risk. In this regard, a better understanding of the mediators and involved signaling pathways is of prime importance.

The nuclear enzyme poly(ADP)-ribose polymerase-1 (PARP1) is the primary subtype of a protein family, which contains polyadenosine diphosphate ribose and polymerase activity (21). PARP1 is a key moderator for cell death in oxidative stress, ischemia, and excitotoxicity (21, 22). PARP1 utilizes oxidized nicotinamide adenine dinucleotide (NAD+) as a substrate to catalyze the covalent attachment of ADP-ribose units onto various target proteins, such as aspartate, glutamate, lysine, tyrosine, and serine. Moreover, PARP1 catalyzes the addition of NAD+ of poly (ADP)-ribose (PAR) onto itself in response to oxidative DNA damage (21).

Recently, researchers have employed metabolomics analysis to investigate the roles and molecular pathways involved in cellular metabolism disorders under oxidative stress (23, 24). Oxidized NAD (NAD+) and reduced NAD (NADH) as metabolic cofactors play vital role in cellular energy metabolism and are also involved in calcium homeostasis, mitochondrial function, oxidative stress, gene expression, aging and apoptosis (25–27). DNA damage-induced PARP activation leads to depletion of NAD+ which subsequently impedes cellular energy metabolism. Moreover, PARP1 activation could hinder hexokinase (HK), which is a crucial enzyme in the glycolysis pathway through PARylation process and subsequently leading to ATP deprivation and cell death called id parthanatos (28–31).

The signaling pathways involved in the neuronal death induced by PARP1 activation are not yet fully determined. Different studies have been conducted on this regard (29, 32–34).

Enrichment analysis revealed NAD+-related metabolic disorders induced by excessive activation of PARP1 after DNA damage (35). Activating PARP1 would lead to cytosolic NAD+ depletion and mitochondrial release of apoptosis-inducing factor (AIF), and different studies have investigated the causal relationships between PARP1 activation and NAD+ depletion. Strong evidence shows that NAD+ depletion is a causal process in PARP1-mediated cell death so that NAD+ depletion and glycolytic failure result in mitochondrial AIF release (36).

Conrad et al. showed that NAD+ depletion is necessary and sufficient for PARP1-mediated neuronal death (36). They used extracellular NAD+ to restore neuronal NAD+ levels after PARP1 activation. Exogenous NAD+ used P2X (6) -gated channels to enter neurons to restore cytosolic NAD+ that subsequently inhibited excess PARP1 activation and prevented the AIF translocation, glycolytic inhibition, mitochondrial failure, and neuron death. They used metabolic substrates, such as pyruvate, hydroxybutyrate, or acetoacetate to circumvent the glycolytic inhibition and then prevented mitochondrial failure and neuron death. Other finding of this group was that using NAD+ glycohydrolase to deplete intracellular cytosolic NAD+ lead to blockage of the glycolysis inhibition, AIF translocation, mitochondrial depolarization, and neuron death, and the process was independent of PARP1 activation (36).

NAD+ is an important coenzyme in redox reaction of cells and plays significant roles in the process of cell tricarboxylic acid cycle (TCA), fat β oxidation, glucose metabolism, and amino acid metabolism (37). Recent studies have confirmed that excessive PARP1 activation could promote NAD+ depletion, which could affect cell energy metabolism and reduce ATP levels leading to cell necrosis (22). Some studies have investigated the causal relationship of PARP activation and subsequent NAD+ depletion and cell death and demonstrated direct evidence on causal relationship between PARP activation, NAD+ depletion, and cell death (38–41). However, later studies have demonstrated that excessive PARP activation and NAD+ depletion is not the only pathway to cell death. Heller et al. used islet cells from mice with a disrupted and inactivated PARP gene (PARP^−/−^ mice) to investigate the effects of DNA-damaging radicals and relationship between PARP activation, NAD+ depletion, and cell death. They reported that mutant islet cells showed more resistant to the toxicity of DNA-damaging radicals and did not show NAD+ depletion after exposure to the DNA-damaging radicals (41). This finding indicates that most of NAD+ depletion following the exposure to oxidative factors is due to PARP activation. They also reported that 3-aminobenzamide, an ADP-ribosylation inhibitor, partially protected islet cells with intact PARP gene but not PARP^−/−^ cells from lysis following nitride oxide or ROI treatment. This finding confirms that the protective action of 3-aminobenzamide is only due to PARP inhibition. They also observed that the mutant cells underwent an alternative pathway of cell death that did not require PARP activation and NAD+ depletion. They confirmed the causal relationship of PARP activation and subsequent islet cell death and concluded there is an alternative pathway of cell death independent of PARP activation and NAD+ depletion (41). Few studies have reported that pulmonary fibroblasts from the PARP^−/−^ mice are protected against peroxynitrite-induced cell injury, in comparison to the fibroblasts of the corresponding wild-type animals (42). Furthermore, Eliasson et al. demonstrated that neural cells of PARP^−/−^ mice show significant protection against different oxidants inducing glutamate-mediated ischemic injuries indicating the involvement of PARP activation in neuronal damage following focal cerebral ischemia (43). The resistance of inactivated PARP gene to different oxidative factors has been reported in different diseases including diabetes. For instance, Burkart et al. showed *PARP*^−/−^ mice are completely resistant to the development of diabetes induced by the beta-cell toxin streptozocin (44). The findings of the previous studies have suggested that PARP1 inhibitors might have protective effects against oxidative stress-induced cell necrosis.

This study aimed to investigate the effects and possible mechanisms of action of ozone on SCNs metabolism using metabolomics analysis. The findings show that NAD+ depletion is caused by excessive activation of PARP1. Moreover, we found that ozone-induced DNA damage could be one of the main causes of ozone-induced metabolic disorders of SCNs. Using PARP1 inhibitors can prevent NAD+ depletion and promote cell viability during ozone exposure.

## Materials and Methods

### Animals and Reagents

The Wistar neonate rats used in this experiment were obtained from the Experimental Animal Center of Shandong University, Shandong, China. The 24-h born Wistar neonate rats were selected for the experiments and treated according to regulations of the National Institutes of Health and all the experimental procedures of this study were approved by the Animal Protection and Use Committee of School of Medicine of Shandong University, Jinan, Shandong, China. During the experiment, efforts were made to reduce the pain caused by the operation.

The cultivating materials including neurobasal medium, DMEM/F12, medium B27 supplement, fetal bovine serum, and trypsin were purchased from Gibco BRL, Life Technologies (Scotland, UK). The agents for laboratory assessments including Poly-L-lysine, L-glutamine, penicillin, streptomycin, ABT-888, cytarabine, protease inhibitors, phenylmethylsulfonyl fluoride (PMSF) were purchased from Beyotime, Beyotime Biotechnology (China).

### Isolation and Cultivation of SCNs

The extraction and isolation and cultivation of SCNs from neonatal rats were performed as per the method described previously with slight modifications (45). Briefly, the 24-h newborn rats were obtained and sacrificed by cervical dislocation under general anesthesia, then immersed in 75% alcohol and iodophor for 5 min, respectively. The spinal cord tissue was isolated and cut into small pieces of ~1 × 1 mm^3^. The micro-slices were then blocked for digestion through moving into digestion medium containing 1 ml 0.25% trypsin in 37°C for 10–30 min. Fifty microliter of fetal bovine serum was used to terminate digestion. The cells were separated from tissues carefully and then centrifuged at 1,000 rpm for 5 min. The supernatant was taken and the sediment was re-suspended in DMEM/F12 medium containing 10% fetal bovine serum and penicillin and streptomycin with the final concentration of 100 U/ml and 0.1 mg/ml, respectively. Then, the cell density was adjusted to 5 × 105 /ml. The cells were inoculated into a 6-well culture plate coated with 0.1 g/L of Poly-L-lysine and cultured in an incubator containing 5% carbon dioxide at 37°C. Six hours later, the whole amount of medium was replaced with neuron-specific culture medium (containing 2 mmol/L L-glutamine, 2% B27 additive, final concentration of 100 U/ml and 0.1 mg/ml penicillin and streptomycin, respectively). After the medium was changed for 24 h, the solution was changed in half once every 2 days, and cytarabine with the final concentration of 0.05 mg/ml was added on the third day to inhibit the growth of glial cells. The cells could be used for subsequent experiment 6–8 days after plating.

### Immunofluorescence Identification

The isolated SCNs were cultured in 96-well plates with glass bottom for 7 days. The medium was absorbed completely. The cells were fixed with paraformaldehyde for 15 min, permeabilized for 5 min at the room temperature with 0.5% Triton X-100, and blocked with goat serum at room temperature for 1 h. The cells were incubated with anti-NF200 (Boster, BM0100, 1:100) overnight at 4°C. On the second day, the cells were incubated with the secondary antibody (Beyotime, A0216, 1:400) at room temperature for 1 h. The 4′,6-diamidino-2-phenylindole (DAPI) counterstain was used to show the chondrocyte nuclei. The stained SCNs were observed under a fluorescence microscope.

### Ozone Exposure and Drug Treatment

The SCNs were exposed to ozone with different concentrations in a computer-controlled external exposure chamber, and the ozone concentration was monitored with an ozone analyzer (model 400 A, Advanced Pollution Instrumentation, San Diego, CA), implemented in the exposed chamber, and connected to a computer for monitoring and adjustment. After ozone treatment, subsequent experiments were performed after washing the samples with PBS for three times. The final concentration of ABT-888 was 10 um. The SCNs were treated with diluted ABT-888 2 h before ozone exposure.

### Cell Viability Assessment

Cell viability was detected by using a Cell Counting Kit-8 (CCK-8) (Beyotime Biotechnology, China). The assessment was performed according to the instructions of the manufacturer. Briefly, the cell density was adjusted to 2 × 10^5^ and the cell suspension was inoculated in a 96-well plate (100 μl/well). The plate was pre-incubated in a humidified incubator (at 37°C, 5% CO_2_) and ABT-888 was added to the wells, then the plate was exposed to ozone after 2 h. Then, 1 h later, 10 μl of the CCK-8 solution was added to each well of the plate slowly. The plate was incubated for 4 h in the incubator. Finally, the absorbance of the sample was measured at 450 nm using a microplate reader. The cell survival rate was normalized to the untreated control group.

### Measurement of ATP Level

ATP levels were detected by using an ATP assay kit (Beyotime Biotechnology, China). According to the instructions provided by the manufacturer, the cells were first lysed by lysis buffer, which was then centrifuged at 12,000 rpm for 5 min at 4°C, and the supernatant was then collected for further assessments. The protein concentration was determined using a BCA kit (Beyotime Biotechnology, China), and then 100 μl of ATP detection working solution was added to the 96-well plate, which was placed at room temperature for 5 min. Then, 100 μl sample or standard solution was added to each well. The RLU value was determined by a chemiluminometer, and the ATP concentration was determined to refer to the RLU standard curve determined by the standard in the kit. Finally, the ATP level was calculated as per the following Equation (1):

(1) $$\begin{array}{r} \text{ATP level}=\text{ ATP concentration}/\text{ total protein concentration} \end{array}$$

### LC-MS Analysis

LC-MS is the most widely used metabolomics analysis thanks to its adaptable components including the ionization technique, stationary, and mobile phases (46, 47). The SCNs were collected into a centrifuge tube and centrifuged at 3,500 rpm for 10 min. The cells were re-suspended by adding 0.5 ml ultrapure water. Then 0.5 ml of low-temperature methanol (−20°C pre-cooled for 6 h) was added and centrifuged at 3,500 rpm for 10 min at 4°C. The supernatant was absorbed and the sample was placed into a 1.5 ml EP tube to be stored at −80°C. Before the test, the sample was carefully thawed on ice. Then, 10 μL of internal standard (2.8 mg/mL, chlorophenylalanine) was added to the sample, which was ultrasonically extracted at 4°C for 30 min, silenced at −20°C for 1 h, centrifuged at 12,000 rpm for 15 min at 4°C, and transferred into the injection vial for testing after 200 μL of the supernatant was removed. Analysis platform was as follows: LC-MS (Thermo, Ultimate 3000LC, Q Exactive). Column: Hyper gold C18 (100 × 2.1 mm 1.9 μm). The chromatographic separation condition was as follows: Column temperature: 40°C; Flow rate: 0.3 mL/min; Mobile phase A: water + 5% acetonitrile + 0.1% formic acid; Mobile phase B: acetonitrile + 0.1% formic acid; Injection volume: 10 μL; Automatic injector temperature: 4°C. ESI+: Heater Temp 300°C; Sheath Gas Flow rate, 45 arb; Aux Gas Flow Rate, 15 arb; Sweep Gas Flow Rate, 1 arb; spray voltage, 3.0 KV; Capillary Temp, 350°C; S-Lens RF Level, 30%. ESI–: Heater Temp 300°C, Sheath Gas Flow rate, 45 arb; Aux Gas Flow Rate, 15 arb; Sweep Gas Flow Rate, 1 arb; spray voltage, 3.2 KV; Capillary Temp,350°C; S-Lens RF.

### Western Blot Assessment

The SCNs were lysed with a cell lysate containing PMSF and phosphatase inhibitors. The cell lysates were collected and centrifuged at 14,000 rpm for 5 min. The supernatants were taken and the protein concentration was determined using a BCA kit (Beyotime Biotechnology, China). The protein sample was boiled for 10 min before loading, and then electrophoresed using 10% SDS gel, transferred to a PVDF membrane, blocked with 5% skim milk at room temperature for 1 h, and then the membranes were incubated overnight at 4°C with anti-PARP1(Abcam, ab151794, 1:1,000 dilution), anti-PAR(CST, #83732, 1:1,000 dilution), anti-γH2AX(CST, #2577, 1:1,000 dilution), anti-GAPDH(CST, #5174, 1:1,000 dilution), anti-p-AMPK (CST, #2535, 1:1,000 dilution), and anti-AMPK (CST, #2532, 1:1000 dilution) primary antibodies in dilution buffer. The cells were incubated with the horseradish peroxidase (HRP)-conjugated anti-rabbit IgG (Beyotime Biotechnology, A0216, 1:1,000 dilution). The membranes were developed using the enhanced chemiluminescence substrate LumiGLO (Millipore, Bedford, MA, USA).

### NAD Assay

The NAD+ concentration was determined by using the NAD/NADH Assay Kit (Abcam, ab65348) according to the instructions of the manufacturer. This assessment was performed as follows. The cells were collected by cell scraping, washed with pre-cooled PBS three times, lysed with the extracted buffer solution and centrifuged with the centrifugal machine at 12,000 rpm for 5 min at 4°C. The supernatant was collected. First, the protein concentration was determined using a BCA kit (Beyotime Biotechnology, China), and then samples were heated at 60°C for 30 min so that NAD+ in the sample could decompose completely. Then, 50 μl of both samples were taken and mixed with 100 μl Reaction Mix and placed at room temperature for 5 min. Then, 10 μl of NADH Developer was added to each well and placed at room temperature for 2 h. The OD450 was measured with a multi-functional microplate reader.

### Statistical Analysis

All data were expressed as Mean ± Standard Deviation (SD). The data were sorted and checked and then the data matrix was imported into SIMCA-P 13.0 (Umetrics AB, Umea, Sweden) software for multivariate statistical analysis. Statistical package for social sciences (SPSS) (Version 20, Windows, IBM Statistics, Chicago, IL, USA) was used for data analyses. Differences among the groups were compared by one-way analysis of variance (ANOVA), then followed by Tukey multiple comparison test or Bonferroni test for pairwise comparison. Differences were considered to be significant at *p* < 0.05. In all experiments, the assessments were repeated at least three times in an independent protocol and the average values were calculated and considered for further analysis. The LC-MS test data were extracted and preprocessed using the Compound Discoverer software (ThermoFisher Scientific, USA), which was finally compiled into two-dimensional data matrix format, including Retention time (RT), molecular weight (CompMW), observation volume, number of substances to be extracted, and peak intensity.

## Results

### Morphological Observation and Identification of Cultured SCNs

**Figure 1:**
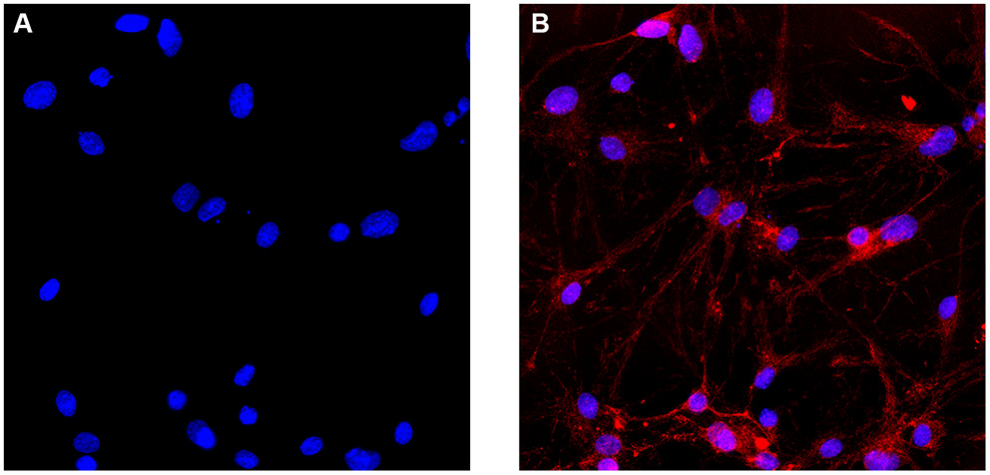
Immunofluorescence identification of SCNs. **(A)** Cell nuclei are identified with DAPI. **(B)** SCNs are stained with the anti-NF200 monoclonal antibody. More than 90% of the cultured cells show positive expression of NF200 on the day 7 of *ex vivo* culture. The number of NF200-positive neurons are counted per area and expressed as percentage of total number of cells (Scale bar: 40 μm).

Cells were observed by inverted phase-contrast microscopy 4 h after seeding in 6-well culture plates. It was observed that most of the cells were attached to the wall of the culture plate. The cells were round and transparent, with good refractive index and stereoscopic effect. A part of the nuclei can be clearly observed. Moreover, 24 h after the seeding, a large number of cells were found to have protrusions, the length of which accounted for about 1/2 of the cell. Forty eight hours after the seeding, the number of protruding cells increased, accounting for about 1/3 of the total number of cells, and the length of the protrusion increased. Ninety six hours after the seeding, more cell debris was observed under the microscope, which was caused by the addition of cytarabine inhibiting the glial cells on the third day of culture. On the 7th day of culture, the cells were aggregated slightly, and the cell protrusions grew obviously. The neurons and their protrusions were connected and formed a network. On the 7th day of culture, immunofluorescence staining with an anti-NF200 monoclonal antibody was performed. The results showed that more than 90% of cells show positive expression of NF-200 (Figures 1A,B).

### Ozone Exposure and SCNs Viability

**Figure 2:**
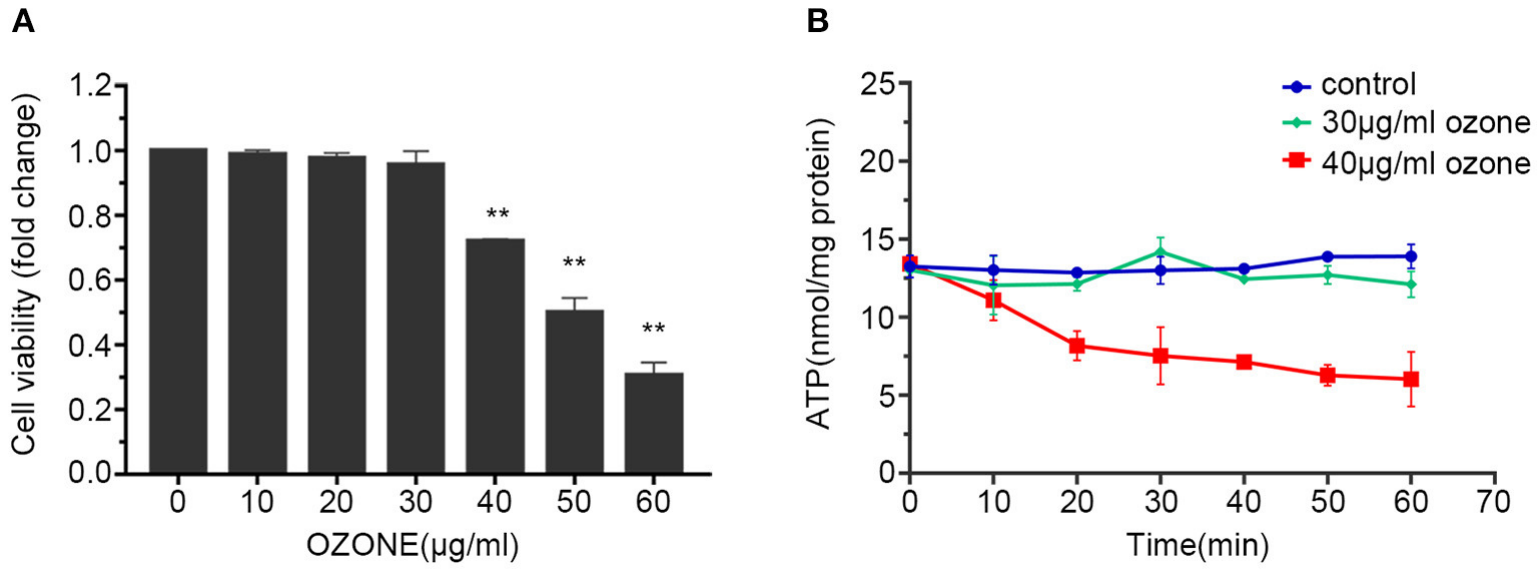
Ozone decreases the viability and ATP levels of SCNs. **(A)** SCNs are exposed by ozone at 10, 20, 30, 40, 50, 60 μg/ml concentrations for 1 h, and cell viability was measured by CCK-8 assay. **(B)** The ATP levels of SCNs are determined by ATP assay kit after 30 and 40 μg/ml ozone exposure for 10–60 min. Data are presented as Mean ± SD of three independent experiments. **P < 0.01, compared with the control.

In the majority of the ozone therapy for LBP, the common concentration of the ozone is 30 μg/ml, and some studies have tried to use a concentration of 40 μg/ml or even higher (4, 6, 7, 14, 48, 49). Therefore, when observing the influence of ozone on the viability of SCNs, 10, 20, 30, 40, 50, and 60 μg/ml ozone were applied. The results showed that the cell viability of the SCNs decreased with the increase in ozone concentration. The cell viability of 10, 20, 30 μg/ml ozone treatment groups were not significantly different from the control group, while the viability of SCNs in the 40, 50, 60 μg/ml ozone treatment groups were significantly lower than the control group. The viability of SCNs in the 40 μg/ml ozone treatment group was 71.91%, and the cell viability in 50 and 60 μg/ml ozone treatment groups was 49.97 and 30.43%, respectively (Figure 2A). Since 50 and 60 μg/ml concentrations are not recommended in the most clinical applications, 30 and 40 μg/ml concentrations were used for further subsequent experiments.

### Ozone Exposure and ATP Level of SCNs

Previous studies have found that ozone exposure can reduce intracellular ATP level (50–52). Therefore, the influences of 30 and 40 μg/ml concentrations on the energy metabolism index (ATP level) of SCNs were studied first. Our results showed that the groups received 30 and 40 μg/ml doses of ozone, 15 min after the exposure the intracellular ATP level significantly decreased compared with the control group. After that, the ATP level of 30 μg/ml ozone exposure group recovered gradually, whereas the 40 μg/ml ozone exposure group showed further rapid decline in the ATP level. After 60 min, the 40 μg/ml ozone exposure decreased the ATP level of SCNs compared with the control and 30 μg/ml ozone exposure groups (Figure 2B).

### Ozone Exposure and Metabolic Disorder of SCNs

**Figure 3:**
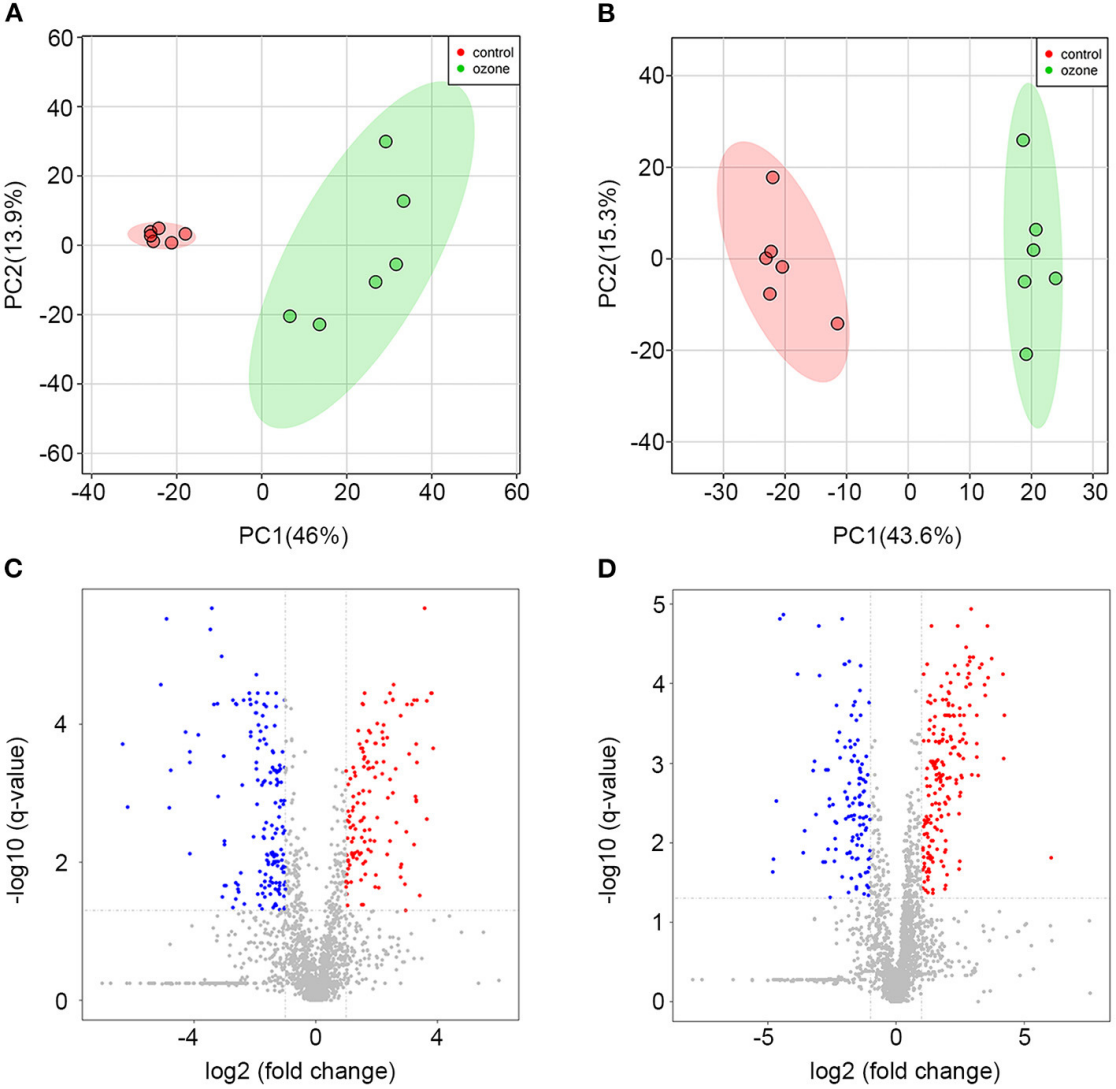
Metabolomics profiling analyses for 40 μg/ml ozone group vs. control group. **(A)** Score plots of the principal component (PC) distinguishing 40 μg/ml ozone group (*n* = 6) from control group (*n* = 6) based on their metabolomics pattern with PC-1 (46.0%) and PC-2 (13.9%) in ESI+ mode. **(B)** Score plots of the PC distinguishing 40 μg/ml ozone group from control group based on their metabolomics pattern with PC-1 (43.6%) and PC-2 (15.3%) in ESI- mode. **(C,D)** Volcano plot demonstrates metabolite changes in ESI (±) mode. Fold change (FC) on the x-axis and FDR-adjusted *P*-values on the y-axis. Black vertical and horizontal lines show the filtering criteria (FC = 1.0 and FDR corrected *P*-value < 0.05).

**Figure 4:**
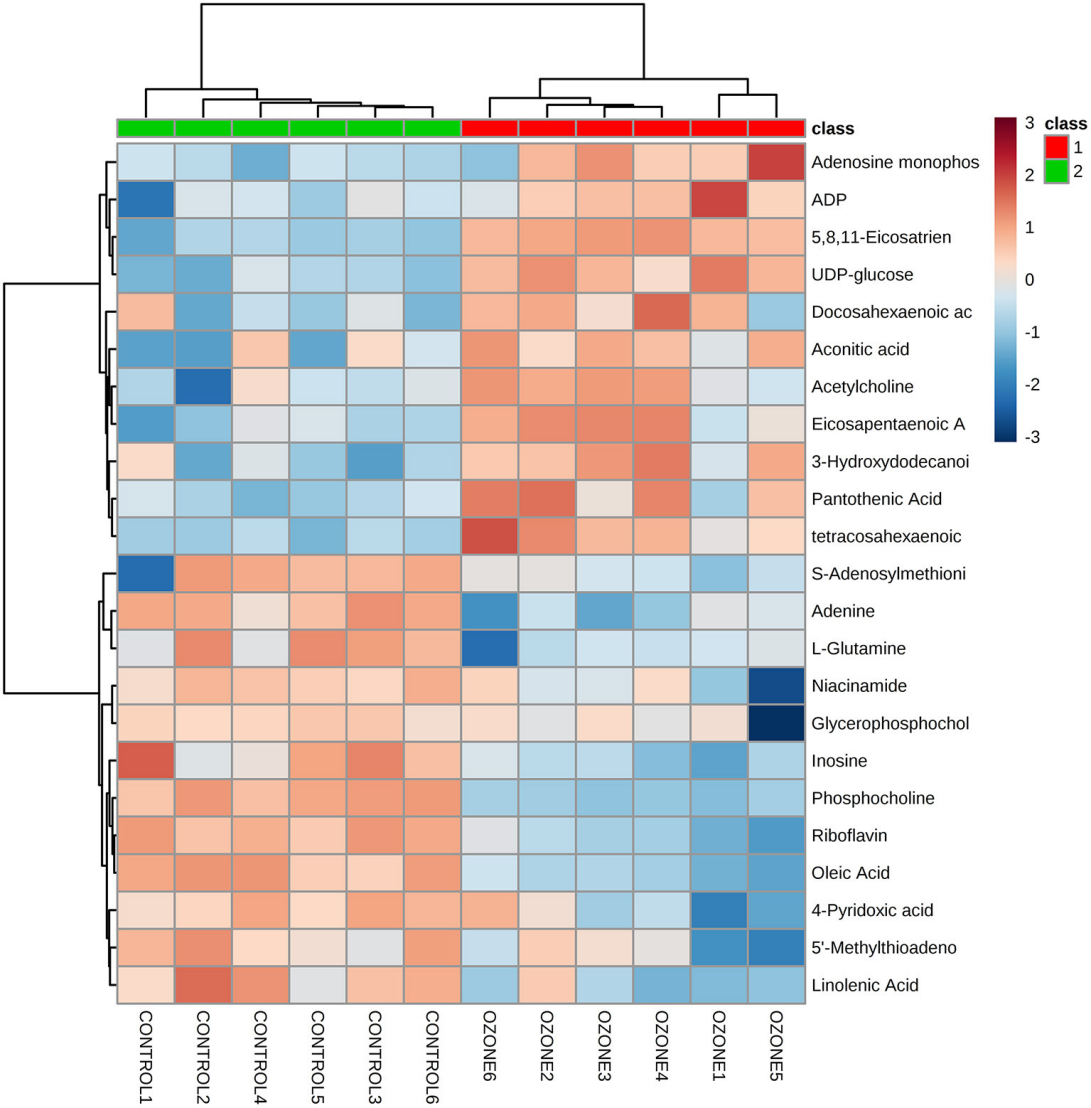
Heat maps shows a comparison between 40 μg/ml ozone group and control group using normalized intensities of 23 significant metabolites. Two distinct clusters are identified. Orange and blue, respectively indicate increased and decreased expression compared with control groups.

To explore specific influences of ozone on the metabolism of SCNs, non-targeted LC-MS metabolomics technique was employed in this study. The SCNs of 40 μg/ml ozone exposure group and the control group were analyzed by non-targeted LC-MS technique to obtain ion chromatogram in Electrospray ionization (ESI) (±) mode, and the LC-MS data were extracted and pre-processed with Compound Discoverer software to obtain a data matrix consisting of information, such as retention time, accurate molecular weight and peak intensity, including a total of 2,268 (ESI+) and 2,210 (ESI–) features. Unsupervised PCA analysis results showed that PC1 in positive mode contained 46.0% cumulative variance, followed by PC2 13.9%, while PC1 in negative mode, contained 43.6% cumulative variance, followed by PC2 15.3% (Figures 3A,B). Abundance metabolites with significant differences between the 40 μg/ml ozone exposure group and the control group were determined through the volcano plot (Figures 3C,D). Then, different substances between different groups were clustered and analyzed and the thermograms were used to reflect relative changes of 23 important metabolic molecules. The most affected pathways were related to fatty acid, tricarboxylic acid cycle, purine metabolism, niacin and nicotinamide metabolism, amino acid, lipid metabolism, and riboflavin metabolism pathway (Figure 4). In these differentially expressed metabolites, it was observed that after exposure to 40 μg/ml ozone for 1 h, the levels of ADP and AMP in the SCNs increased (Figure 4). Moreover, our study showed that the level of ATP in the SCN cells decreased after ozone exposure. Generally, the ratio of ATP/ADP and ATP/AMP in cells could reflect the energy metabolism of cells. Decrease in the ratio indicated the abnormal energy metabolism of SCNs after ozone exposure (53). The intracellular synthesis pathway of NAD+ mainly includes *de novo* production and salvage pathways (54). Niacinamide is a precursor molecule of NAD+ salvage synthesis, whose level decreased after ozone exposure, which indicated that the intracellular NAD+ salvage synthesis pathway was activated (Figure 4). Moreover, levels of aconitine and UDP-glucose were elevated, indicating the utilization of cellular glucose and TCA circulatory disorders (Figure 4). Meanwhile, a series of abnormal changes of pathways including fatty acid metabolism, amino acid metabolism, and lipid metabolism reflected the abnormal metabolism of SCNs under 40 μg/ml ozone exposure (Figure 4). It is worth noting that the decrease of NAM levels and ATP/AMP, ATP/ADP ratios suggests that intracellular NAD+ is consumed excessively, resulting in metabolic disorder.

### Ozone and DNA Damage, PAPR1 Excessive Activation, and NAD+ Depletion

**Figure 5:**
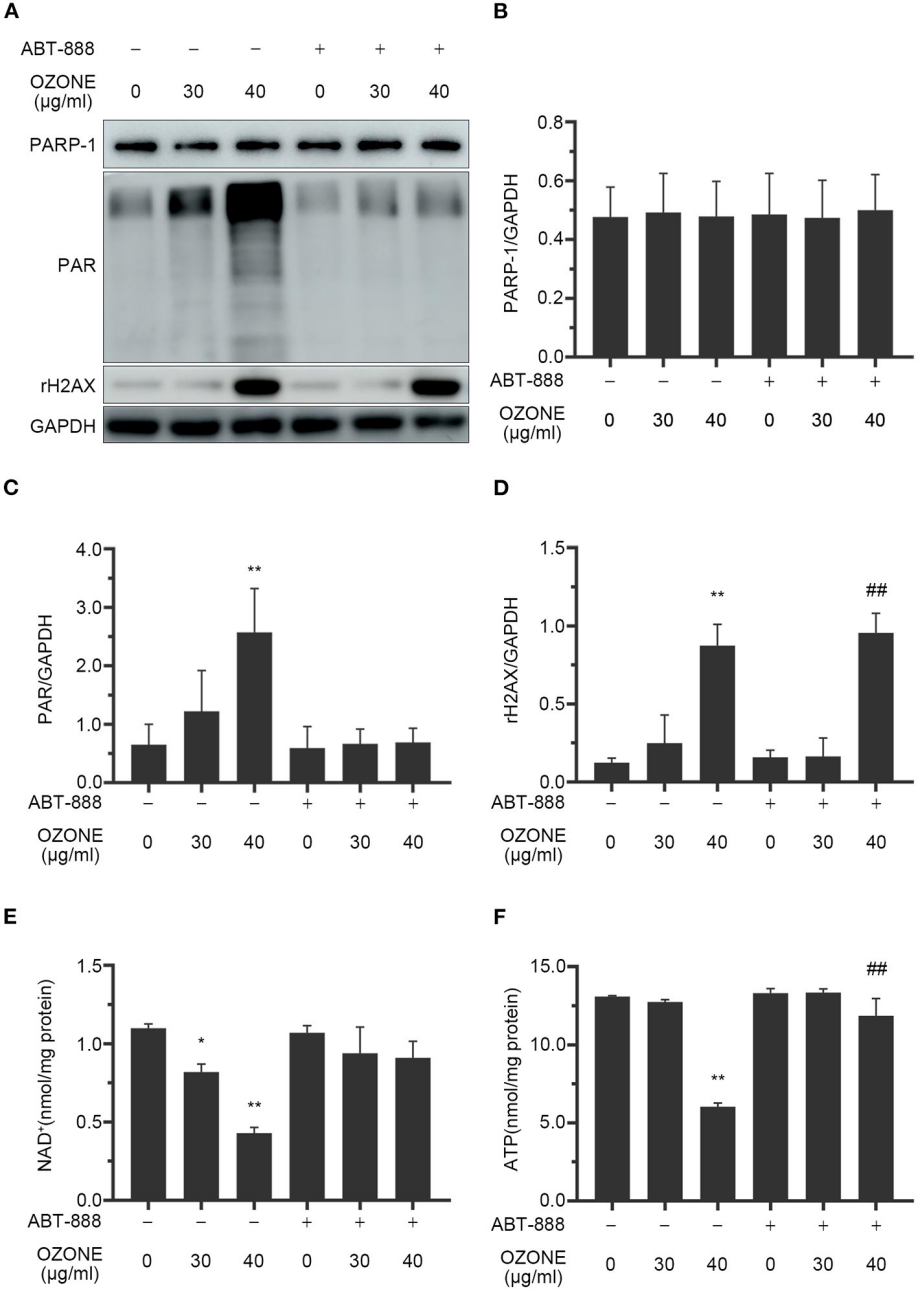
Ozone causes DNA damage, PAPR-1 excessive activation, NAD+ depletion and decline of ATP levels of SCNs. ABT-888 inhibits the NAD+ and ATP declines by inhibiting PARP1 excessive activation. **(A–D)** SCNs are treated with ozone (30 and 40 μg/ml for 60 min) in the absence or presence of ABT-888. The levels of PARP1, PAR, and γH2AX are measured and quantified by western blot. **(E,F)** Ozone exposure decreases the levels of NAD+ and ATP, which are prevented by ABT-888 pretreatment. Data are expressed as Mean ± SD of three independent experiments. *P < 0.05, **P < 0.01, compared with the control without ABT-888 pretreatment; ^##^*P* < 0.01, compared with control with 40 μg/ml ozone without ABT-888.

Studies have reported that excessive activation of PARP1 by cellular DNA damage under oxidative stress can lead to NAD+ depletion and a decrease in ATP levels. Strong evidence confirms that ozone can cause DNA damage (51, 55–57). Therefore, γH2AX, PARP1, PAR, NAD+, and ATP levels are measured to evaluate the toxic effects of 30 and 40 μg/ml ozone on SCNs. The results demonstrated that the levels of DNA damage marker protein in the 30 μg/ml ozone group showed no significant difference with the control group and similarly there was no significant difference between PARP1 levels and PAR levels, compared with the control group (Figures 5A–D). However, the levels of γH2AX in the 40 μg/ml ozone group increased significantly compared with the control group, indicating the presence of a DNA damage in the SCNs (Figures 5A,D). There was no significant difference between PARP1 and the control group, and the PAR levels increased significantly compared with the control group, indicating that PARP1 was excessively activated (Figures 5A–C). As it was expected, the intracellular NAD+ and ATP levels in 40 μg/ml ozone group decreased significantly compared with the control group (Figures 5E,F). These results demonstrate that 40 μg/ml ozone could deplete the NAD+ in SCNs, accompanying PARP1 excessive activation and DNA damage.

### PARP1 Inhibitors and SCNs Protection Against Ozone Exposure and NAD+ Depletion

**Figure 6:**
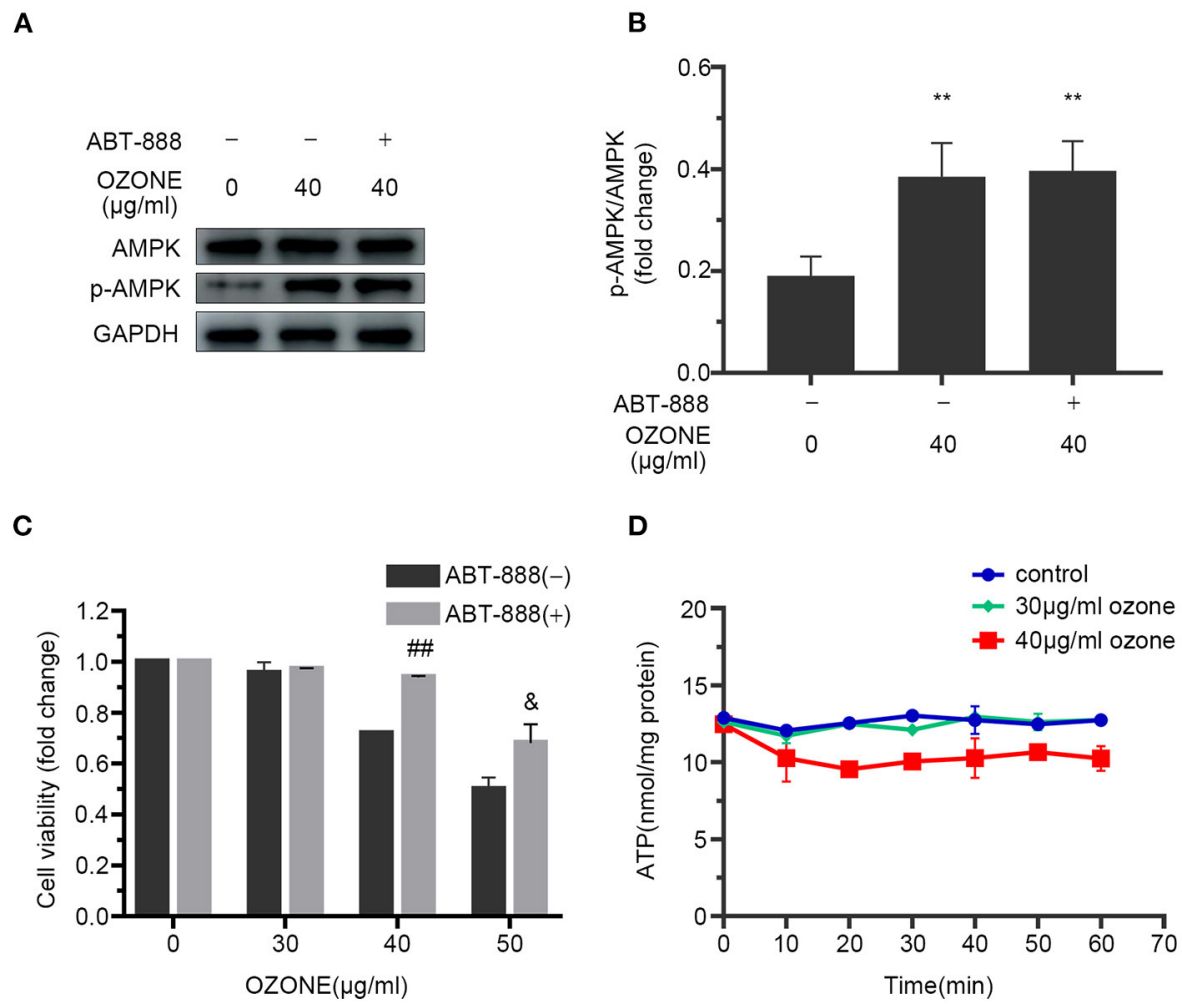
ABT-888 prevents the declines in viability and ATP level of SCNs after 40 μg/ml ozone exposure. **(A,B)** 40 μg/ml ozone exposure activates AMPK of SCNs in the absence or presence of ABT-888. **(C)** ABT-888 improves the viability of SCNs with 40 and 50 μg/ml ozone exposure. **(D)** ATP level is measured after 10–60 min ozone exposure with 30 and 40 μg/ml doses. The data are presented as Mean ± SD of three independent experiments. **P < 0.01, compared with the control; ^##^*P* < 0.01, compared with 40 μg/ml ozone groups without ABT-888 pretreatment; ^&^*P* < 0.05, compared with 50 μg/ml ozone groups without AB.

Many studies have revealed that PARP1 inhibitors could protect cells by preventing NAD+ excessive consumption (22, 58). In this study, we observed the mechanism of ABT-888 alleviating the toxicity of ozone to SCNs. The results showed that after application of ABT-888, the PAR levels of SCNs under 30 and 40μg/ml ozone exposure conditions showed no significant difference with the control group (Figures 5A–C). Moreover, there was no significant decrease in NAD+ and ATP levels of 30 and 40 μg/ml ozone groups, compared with the control group (Figures 5E,F). The viability of SCNs was significantly higher than that of the non-inhibitor intervention groups after 40 and 50 μg/ml ozone exposure, indicating that PARP1 inhibitors could avoid NAD+ depletion by preventing PARP1 excessive activation and exert neuron-protective effects under the ozone exposure conditions (Figure 6C).

## Discussion

This study investigated the cytotoxic effects of ozone exposure on SCNs through determining the induction of the metabolic disorder and the possible involved signaling pathways in these neurons. The metabolic disorder in SCNs induced by ozone exposure was demonstrated by non-targeted LC-MS analysis. Moreover, our findings showed that NAD+ depletion caused by excessive PARP1 activation could serve as the main cause of abnormal cellular metabolism. We also found that PARP1 inhibitors could protect SCNs from ozone exposure through avoiding energy metabolism disorders caused by NAD+ depletion.

Currently, ozone therapy is widely used to treat lumbar disc herniation, soft tissue pain and arthritis in different countries including Germany, Italy, Spain, and China (3). In 2004, Muto et al. conducted a clinical retrospective study on 2,200 patients treated with intervertebral ozone injection. The results showed that the positive rate of treatment of patients with single-segment disc herniation, extensive disc degeneration, and the calcified intervertebral disc was 64, 40, and 25%, respectively (59). In 2012, Francisco et al. conducted a statistical analysis of four previous clinical random cohort studies and eight clinical retrospective observations on ozone treatment of LBP, among which evidence levels for the study of the intervertebral disc and paravertebral ozone injection were II-1 and II-3. The grading of recommendation was 1C for intradiscal ozone therapy and 1B for paravertebral ozone therapy (13). The findings showed that ozone is effective in treating LBP. However, due to the powerful oxidative property of ozone, irregular treatment may injure the nerves and the surrounding tissues. Some cases have reported that ozone treatment of lumbar disc herniation can cause injury to the ventral and dorsal root ganglia (20). However, there are still fewer studies on the effects of ozone on SCNs. Therefore, it is necessary to study thoroughly the influences of ozone exposure on SCNs.

Previous studies have found that ozone exposure can reduce intracellular ATP levels (51). Therefore, in this study, the influences of ozone on the ATP level of SCNs at concentrations of 30 and 40 μg/ml were measured first, and it was found that the intracellular ATP concentration recovered rapidly after a short decline at the concentration of 30 μg/ml. Nevertheless, the intracellular ATP levels in 40 μg/ml ozone group declined continuously.

Subsequently, non-targeted LC-MS analysis was employed to analyze further specific changes of intracellular metabolism of SCNs after 40 μg/ml ozone exposure. The results showed that the expressions of metabolic molecules, such as ADP, AMP, aconitic acid, and UDP-glucose increased, while the molecular expressions of NAM, L-glutamine, and riboflavin were down-regulated. Furthermore, all differentially expressed metabolic molecules included mainly fatty acid, tricarboxylic acid cycle, purine metabolism, niacin and nicotinamide metabolism, amino acid, lipid metabolism, and riboflavin metabolism pathways. First, it can be clarified that the intracellular ATP/ADP and ATP/AMP ratios can reflect the energy metabolism conditions, because as the most basic energy metabolism-related molecule in cells, decrease of its ratio indicates the energy metabolism of SCNs after ozone exposure is disordered (60, 61). NAD+ is an essential coenzyme for redox reaction in cells which actively participates in many physiological reactions, such as TCA, fat beta-oxidation. Therefore, it is of great significance in the metabolic utilization of nutrients, such as sugar, fat and amino acids. As the most important hydrogen donor in the electron transport chain, NAD+ participates in the production of ATP (62). Furthermore, NAD+-related metabolites, such as coenzyme II [NADP (H)], NAM, and ADP ribose played important roles in human cell energy metabolism, oxidative stress regulation and signaling pathway transmission (37). The synthesis pathways of NAD+ in cells mainly include the salvage synthesis pathway using NAM and the *de novo* synthesis pathway using tryptophan. The results of metabolomics analysis demonstrated that NAM levels reduced significantly after ozone exposure, indicating the over-consumption of NAM by salvage pathways of NAD+. Moreover, elevated levels of aconitine and UDP-glucose suggested disorders of cellular TCA cycle and glucose utilization (63). Our study has validated further that ozone could cause a series of differential expressions of metabolic molecules in SCNs, and suggested that the metabolic disorders were associated with excessive consumption of NAD+.

Studies have found that ozone exposure can cause DNA damage in cells and a significant decrease in ATP levels and lead to a reduced Sirtuin3 expression (51). Cheng et al. found that single-cell gel electrophoresis and elevated levels of 8-oxoguanine suggested that ozone exposure induced an increase in DNA single-strand breaks in A549 cells (55). NAD+ is an energy-sensitive metabolic molecule, and the only substrate for the coenzyme I depleting enzymes (PARP, CD38, CD157, SIRT1-SIRT7) and its excessive depletion leads to impaired intracellular energy metabolism (64). However, under oxidative stress, DNA single-strand break in the cells activates PARP1, which leads to depletion of a large amount of NAD+ to repair the damaged DNA. Several studies have shown that the NAD+ depletion caused by excessive activation of PARP1 is an important cause of disorder of cell energy metabolism and cell death (34). In this study, γH2AX, PARP1, PAR, NAD+, and ATP levels are measured to evaluate the DNA damage of cells at 30 and 40 μg/ml ozone concentrations and the influences of the accompanying PARP1 activation on cellular NAD+ and ATP levels. DNA damage marker protein γH2AX increased after ozone exposure, suggesting that ozone exposure could induce DNA damage in SCNs, which is consistent with the findings of the previous similar studies. Further studies have revealed that 40 μg/ml ozone exposure can cause excessive activation of PARP1 in SCNs and cause a decrease of intracellular NAD+ and ATP levels, thereby leading to cell death.

The signaling pathways of DNA damage are vital for the maintenance of genome integrity and dictating DNA repair pathway choice or cell fate decision. PARP1 serves as a rapid sensor for a DNA damage that plays vital roles in driving the early chromatin organization and DNA repair pathway choice at the damage sites. After a DNA damage, PARP1 is rapidly triggered in a damage dose-dependent manner. However, little is known on the influences of PARP1 activation on the damaged cells at the cellular level. Murata et al. used a new combined phasor approach to investigate the effects of PARP1 activation in response to DNA damage on the cellular level (65). They employed fluorescence-based biosensors and fluorescence lifetime imaging microscopy combined with laser micro-irradiation to assess metabolic changes at high spatiotemporal resolution in a living cell (65). They observed that nuclear DNA damage activated PARP-dependent NAD+ depletion, which in turn triggered a rapid cell-wide accumulation of the bound NADH fraction. Their findings showed that PARP activation induces a change in the cellular metabolism that leads to activation of a pro-survival response (65). The NAD+ depletion and NADH accumulation on the cell-wide dimension was accompanied by a metabolic balance shift to oxidative phosphorylation (oxphos) over glycolysis. This study along with the other similar findings discussed that the oxphos inhibition leads to parthanatos due to rapid PARP-dependent ATP deprivation demonstrating that oxphos is a vital factor for recovery and survival of the damaged cell (34, 35, 65).

Several studies have confirmed that PARP1 inhibitors could protect cells from excitotoxicity and DNA damages (57, 65). Zhang et al. reported that PAPR1 inhibitors can counteract the NAD+ depletion caused by atrial fibrillation and alleviate oxidative stress and DNA damage in cardiomyocytes (57). Other studies have demonstrated that PARP1 inhibitors can improve the survival rate of HepG2 cells under oxidative stress, and metabolomics analysis technology has confirmed that its protective effect is related to NAD+ depletion caused by excessive activation of PARP1 (35). However, so far there is no report on the use of PARP1 inhibitors to protect SCNs under ozone exposure conditions. In this study, ABT-888 was employed to inhibit PARP1 activation 2 h before ozone exposure and the results showed that PARP1 activation was inhibited under ozone exposure, while intracellular NAD+ and ATP levels were elevated significantly compared with the control group received no ABT-888 inhibition (Figures 5E,F). Under the exposure conditions of 40 and 50 μg/ml ozone, PARP1 inhibitor increased the viability of SCNs.

Mammalian target of rapamycin (mTOR) signaling pathway incorporates both intracellular and extracellular signals into an integrated central regulator for cell metabolism, growth, proliferation and survival. Some studies have demonstrated that ozone in the dose range used for therapeutic applications exerts different therapeutic effects through AMPK phosphorylation and AMPK/mTOR signaling pathways (11, 66). Evidence shows that reduced autophagy in chondrocytes is the main etiology for articular cartilage degradation and thus development of OA (67–69). Zhao et al. investigated the role of autophagy induction in the therapeutic effects of ozone on osteoarthritis (OA) chondrocytes (11). They reported that ozone improved the decreased level of autophagy in chondrocytes exposed with cytokine interleukin-1β (IL-1β) through activation of the AMPK/mTOR signaling pathway. Moreover, ozone treatment decreased inflammation and restored metabolic balance in the chondrocytes.

Ozone exposure significantly decreased mTOR and P62, whereas increased LC3 II, Beclin-1, and ULK1 proteins in the OA prone chondrocytes. Furthermore, ozone significantly upregulated the levels of p-AMPK, and the improved autophagy in chondrocytes stimulated with IL-1β was suppressed by com C. In addition, ozone treatment significantly suppressed inflammation and regulated metabolism in IL-1β-stimulated chondrocytes. AMPK activation plays a vital role in facilitating autophagy after ozone exposure in chondrocytes stimulated with IL-1β.

However, the specific mechanisms and signaling pathways involved in the activation of AMPK by ozone are not yet fully understood. AMPK serves crucial role in regulating cell energy metabolism and the main factors responsible for AMPK activation are ATP/AMP and ATP/ADP ratios as well as intracellular AMP levels AMPK (70). Our study showed that 30 and 40 μg/ml doses of ozone decreased the ATP levels within 20 min after the exposure (Figure 2B). Moreover, 40 μg/mL ozone significantly increased the levels of ADP and AMP in the SCNs. Previous *in vitro* dose-response studies have shown that ozone in low concentrations (20–40 g/ml of oxygen-ozone) is not toxic to astroglial cells, whereas in higher concentrations (60 μg/mL) significantly decreases cell viability (71). Our findings showed that ozone exposure increased the levels of AMPK phosphorylation in SCNs (Figures 6A,B). However, intracellular ATP levels decreased gradually under 40 μg/ml ozone exposure. Our results in support of previous studies, showed that excessive activation of PARP1 could result in DNA damage and NAD+ depletion, and subsequently reduce cell viability. The use of PARP1 inhibitor improved the viability of SCNs under ozone exposures with dosages of 40 and 50 μg/ml (Figure 6C). Meanwhile, after the application of PARP1 inhibitor, intracellular ATP levels in SCNs still decreased 15 min after ozone exposure. After that, unlike the ABT-888(–) groups, intracellular ATP levels of ABT-888(+) groups recovered gradually and maintained a relatively stable level (Figure 6D). The levels of AMPK phosphorylation showed no significant difference with the ABT-888(–) groups, which might be because the intracellular ATP level was still lower than that of the normal group after the application of PARP1 inhibitor (Figures 6A,B,D). Furthermore, previous studies have shown that ROS could activate AMPK directly by oxidizing its cysteine residues (72, 73). Moreover, liver kinase B1 (LKB1) was the upstream kinase of AMPK, which can also activate AMPK by phosphorylating the 172th threonine on the activation loop of AMPK α subunit (74). Nevertheless, the mechanisms by which ozone activates AMPK in SCNs are still unclear and need further studies.

PARP plays important roles in choosing proper DNA repair pathway and improving its efficiency and cell survival. Moreover, excessive PARP activation regulates metabolism and senescence and also influences DNA repair and both apoptosis and necrosis cell death (21). NAD+ depletion by damage-induced PARP activation could lead to inhibition of cellular energy metabolism, because both NAD+ and NADH are actively involved in metabolic process of cellular energy production (27). Moreover, PARP1 activation inhibits hexokinase (HK), which results in ATP deprivation and subsequent cell death parthanatos (28, 31, 75). Parthanatos cell death needs PAR-dependent nuclear translocation of AIF from mitochondria, however, parthanatos cell death has also been reported independent of AIF. In addition, PARP activation induces intracellular acidification that increases the risk of necrosis cell death (76). Therefore, the downstream effects of PARP activation are complex so that the casual association and exact relationships between the levels of DNA damage and the impact of PARP signaling on energy metabolism and/or triggering cell death is not well-understood.

## Conclusion

This study investigated the cytotoxic effects of ozone exposure at different doses on cell viability, ATP levels and the NAD+ concentration in SCNs in animal model. The findings showed that 40 μg/ml ozone exposure could cause DNA damage and metabolic disorders of SCNs. PARP1 inhibitors could prevent NAD+ depletion caused by excessive activation of PARP1 during ozone exposure and thus alleviate the toxicity of ozone to SCNs without inhibiting the activation of AMPK.

## Data Availability Statement

The raw data supporting the conclusions of this article will be made available by the authors, without undue reservation.

## Ethics Statement

The animals used in this experiment are selected strictly according to relevant regulations of the National Institutes of Health and approved by the Animal Protection and Use Committee of School of Medicine of Shandong University.

## Author Contributions

SM and XZ: conceptualization. SM: methodology, software, formal analysis, resources, writing—original draft preparation, and visualization. ZF, TS, and XL: validation. YL: investigation. CZ and PS: data curation. ZF: writing—review and editing, supervision, project administration, and funding acquisition. All authors contributed to the article and approved the submitted version.

## Conflict of Interest

The authors declare that the research was conducted in the absence of any commercial or financial relationships that could be construed as a potential conflict of interest.

## Abbreviations

LBP: Low back pain
SCNs: spinal cord neurons
LC-MS: liquid chromatography-mass spectrometry
PARP1: poly (ADP)-ribose polymerase-1
NAD+: nicotinamide adenine dinucleotide
AMPK: AMP-activated protein kinase
TCA: tricarboxylic acid cycle
PMSF: phenylmethylsulfonyl fluoride
DAPI, 4′: 6-diamidino-2-phenylindole
RT: retention time
LKB1: liver kinase B1.
